# Recent insights into the molecular mechanism of ubiquinol oxidation by cytochrome *bc*_1_

**DOI:** 10.1002/2211-5463.70340

**Published:** 2026-09-15

**Authors:** Anna Wójcik‐Augustyn, Artur Osyczka, Marcin Sarewicz

**Affiliations:** ^1^ Department of Molecular Biophysics, Faculty of Biochemistry, Biophysics and Biotechnology Jagiellonian University in Kraków Poland

**Keywords:** cytochrome *bc*_1_, electron bifurcation, electron transfer, EMET, proton transfer, ubiquinol oxidation

## Abstract

This review summarizes the long‐standing effort to understand the mechanism of quinol oxidation catalyzed by cytochrome *bc*_1_. Cytochrome *bc*_1_ is an enzyme involved in both the respiratory and photosynthetic electron transport chains. It couples quinol oxidation with quinone reduction at two separate active sites (Q_o_ and Q_i_, respectively) catalyzing proton and electron transfer with high efficiency. At the Q_o_ site, the reaction involves the separation of two electrons derived from the quinol oxidation, which are transferred to opposite sides of the enzyme across the membrane, the so‐called electron bifurcation (EB). Despite many years of studies, the molecular mechanism of EB and the molecular basis of its efficiency remain a matter of debate. Here, we reflect on major issues that remain unsolved and thus hamper understanding of EB. We then discuss new perspectives afforded by recent experimental and computational studies that lead to consideration of a new mechanism of EB. This mechanism, named EMergent Electron Transfer (EMET), proposes a departure from a canonical reaction scheme to explain the high fidelity of EB. Ultimately, the theory of EMET provides a constructive conceptual framework for further experimental explorations.

## Graphical abstract

The review reflects on the mechanism of the catalytic reaction in cytochrome bc_1_: the electron bifurcation that involves the separation of two electrons derived from the quinol oxidation to opposite sides of the enzyme across the membrane.

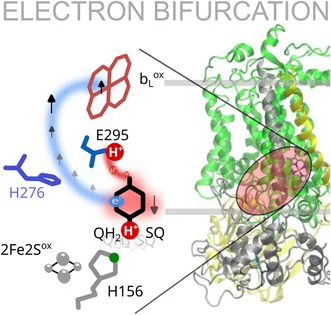

**Abbreviations**
2Fe2S: Rieske [2Fe‐2S] cluster
Cyt*b*
: cytochrome *b*
Cyt*bc*_1_
: cytochrome *bc*_1_
Cyt*c*_1_
: cytochrome *c*_1_
EB: electron bifurcation
*E*_m_
: redox midpoint potential
EMET: emergent electron transfer
ET: electron transfer
HOMO: highest occupied molecular orbital
ISP: Rieske iron–sulfur protein
ISP‐HD: iron–sulfur protein head domain
LUMO: lowest unoccupied molecular orbital
pmf: proton motive force
Q: quinone
QBEB: canonical quinone‐based electron bifurcation mechanism
QH_2_
: quinol
QM: quantum mechanics
QM/MM: quantum mechanics/molecular mechanics
Q_o_, Q_i_
: ubiquinol oxidation and reduction sites, respectively
RMSD: root‐mean‐square deviation
ROS: reactive oxygen species
SC: short‐circuit
SQ: semiquinone, either protonated or anionic
SQ‐2Fe2S: semiquinone spin‐coupled to the reduced 2Fe2S cluster
SQH: neutral semiquinone

## Cytochrome *bc*_1_

**Fig. 1:**
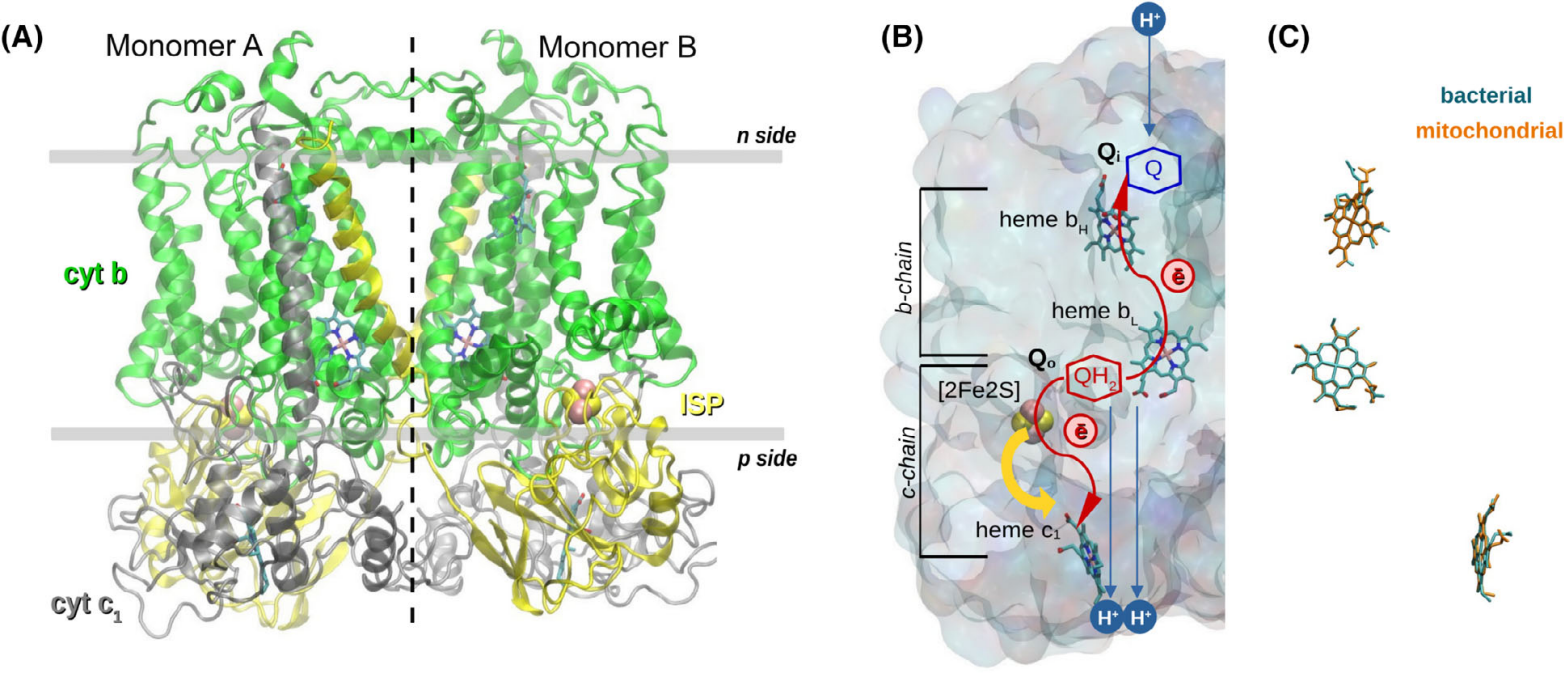
Structure and function of Cyt*bc*_1_. (A) The crystallographic structure of the cytochrome *bc*_1_ (Cyt*bc*_1_) dimer from *R. capsulatus* (PDB code: 1ZRT). Each monomer contains three catalytically active subunits: cytochrome *b* (Cyt*b*, green), cytochrome *c*_1_ (Cyt*c*_1_, silver), and iron–sulfur protein (ISP, yellow). (B) The diagram showing the oxidation of one quinol molecule at the Q_o_ site occurring as a result of electron bifurcation: red arrows indicate the direction of electron transfer; blue arrows indicate the direction of proton transfer; yellow arrow indicate the large‐scale movement of ISP head domain (ISP‐HD). (C) Comparison of the distribution of heme cofactors: *b*_H_, *b*_L_, and *c*_1_ in bacterial (cyan) and mitochondrial (orange) Cyt*bc*_1_. The 2Fe2S cluster was not included in the comparison because it is located in the ISP‐HD domain, which can adopt different conformations depending on the occupancy of the Q_o_ site.

Cytochrome *bc*_1_ (Cyt*bc*_1_), also known as Complex III, is involved in respiration and bacterial photosynthesis. Using two cooperating active sites, where quinol oxidation and quinone reduction occur, Cyt*bc*_1_ is engaged in the electron transport chain and contributes to the generation of the electrochemical proton gradient (*pmf*). Structurally it is a transmembrane homodimer where each monomer contains the catalytically active core of three subunits: Cyt*b*, Cyt*c*_1_ and ISP (Rieske iron–sulfur protein) (Fig. 1A). While some bacteria (e.g., *Paracoccus denitrificans*, *Rhodospirillum rubrum*, or *Rhodobacter capsulatus*) contain only this catalytic core, in others, especially higher, organisms Cyt*bc*_1_ may contain extra subunits that are not directly involved in the catalysis [1, 2, 3, 4, 5, 6, 7]. The three catalytic subunits bind redox‐active prosthetic groups: two *b*‐type and one *c*‐type hemes in Cyt*b* and Cyt*c*_1_, respectively, and a Rieske type 2Fe2S cluster in ISP. The Cyt*b* subunit is composed of eight transmembrane helices from which an evolutionarily conserved four‐helix‐bundle embeds two hemes *b* [2, 3, 8, 9]. The hemes contain low‐spin iron ions coordinated by two conserved histidines provided by two helices from the four‐helix‐bundle [1, 2]. These hemes are the redox‐active elements in quinone‐binding sites and differ in redox midpoint potentials (*E*_m_). The quinone (Q) undergoes reduction at the Q_i_ site, located in the vicinity of the higher potential heme *b*_H_ (*E*_m_ ~ −200 to ~100 mV). Oxidation of quinol (QH_2_) is catalyzed at the Q_o_ site with involvement of the lower potential heme *b*_L_ (*E*_m_ − 290 to −50 mV) [3, 10, 11, 12]. The difference between the *E*_m_ values of heme *b*_H_ and *b*_L_ is maintained among organisms, indicating an evolutionarily conserved direction of ET from the Q_o_ to the Q_i_ site [13, 14]. The Q_i_ site is located on the electronegative (n‐side) and is entirely composed of residues of Cyt*b*, which mediate proton delivery to the site. The Q_o_ site is on the electropositive (p‐side) of the membrane and releases protons to the bulk water [5, 10]. The Q_o_ and the Q_i_ sites are connected via hemes *b*_L_ and *b*_H_ through the pathway named the *b*‐chain (Fig. 1B).

The ISP and Cyt*c*_1_ subunits are composed of single transmembrane helix and hydrophilic globular domain binding prosthetic group: the Rieske cluster or heme *c*_1_, respectively. The ISP helix is anchored in monomer A and its head domain (ISP‐HD) housing the cluster is involved in binding and oxidation of QH_2_ at the Q_o_ site of the monomer B (Fig. 1A) [10, 15]. The adoption of the catalytically active conformation of QH_2_ within the Q_o_ site depends on its interaction with ISP [16]. The available structures of Cyt*bc*_1_ with inhibitors at the Q_o_ site revealed that ISP‐HD may adopt different positions interacting either with Cyt*b* or with Cyt*c*_1_ subunit, which suggested that movement of ISP‐HD is required for effective electron transfer from the Q_o_ site to heme *c*_1_ from Cyt*c*_1_ (this pathway is referred as the c‐chain) [10, 15, 17]. This hypothesis was later confirmed by mutagenesis studies, light‐activated electron transfer (ET) measurements and EPR spectroscopy [18, 19, 20, 21, 22, 23, 24, 25, 26, 27, 28, 29]. The cluster contains two ferromagnetically coupled high‐spin iron ions coordinated by two conserved cysteines and two conserved histidines, respectively [15, 17, 30, 31]. This type of coordination is characteristic for Cyt*bc*_1_ and some oxygenases [32, 33]. Cyt*c*_1_ is anchored by its transmembrane helix on the surface of Cyt*b* within the monomer from which it accepts an electron via ISP‐HD. The electron from the reduced 2Fe2S is accepted by heme *c*_1_, which is covalently bound at the edge of the globular domain of Cyt*c*_1_ by its vinyl moieties with two conserved cysteines (CXXCH motif) [1, 15, 17]. The low‐spin iron ion of heme *c*_1_ is coordinated by conserved histidine and methionine residues [1, 34] and has *E*_m_ comparable to *E*_m_ of 2Fe2S (~100 to ~350 mV) [11, 12, 34]. It is worth noting that in both bacterial and mitochondrial Cyt*bc*_1_, the prosthetic groups within the catalytic core are spatially arranged in an identical manner, giving all‐atom RMSD, computed for distribution of hemes *b* and *c*, equal to 4.44 Å (3.00 Å for position of iron ions, Fig. 1C), which indicates that the optimal arrangement of redox cofactors is evolutionarily conserved, being crucial for effective catalysis.

## The Q cycle

The principles of the Q‐cycle were originally proposed by Mitchell and further modified by Crofts et al. [35, 36]. It considers coupling of QH_2_ oxidation and Q reduction reactions with electron transfer to Cyt*c* and translocation of protons across the membrane. Protons are taken up at the Q_i_ site and used in the reduction of Q to QH_2_, which carries them across the membrane to the Q_o_ site, where they are released upon QH_2_ oxidation (Fig. 1B). The redox reactions at the Q_i_ and the Q_o_ sites are coupled by an electron bifurcation (EB) catalyzed at the Q_o_ site that recycles one of the electrons from QH_2_ and passes it to the Q_i_ site. The second electron derived from QH_2_ is transferred by 2Fe2S to heme *c*_1_ to finally reach diffusible Cyt*c*. This process is associated with a large‐scale movement of the ISP‐HD, which loses its interaction with Cyt*b* in favor of interaction with Cyt*c*_1_ [20, 27, 37, 38, 39, 40, 41]. Consequently, the QH_2_/Q oxidation–reduction process results in proton translocation, contributing to *pmf*. Since the Q_o_ site is the only electron source for the Q_i_ site, the reduction in one quinone molecule at the Q_i_ site requires the oxidation of two quinol molecules at the Q_o_ site [36]. This way, two protons are taken from the n‐side of the membrane and four protons are released at the p‐side, which is associated with transfer of two electrons by Cyt*c* to Cyt*c* oxidase or photosynthetic reaction center in some bacteria [42].

## Electron bifurcation

The Q‐cycle is a mechanism that couples QH_2_/Q oxidation/reduction at the Q_o_ and the Q_i_ sites, respectively. The two‐electron oxidation of QH_2_ at the Q_o_ site involves the process of EB, with involvement of two different electron acceptors of *b*‐ and *c*‐chain, respectively: heme *b*_L_ and 2Fe2S cluster (Fig. 1B). From heme *b*_L_, an electron is transferred via heme *b*_H_ to the Q_i_ site, while 2Fe2S cluster undergoes a large‐scale movement to facilitate ET to heme *c*_1_. Although the EB process is very efficient, theoretical considerations have been struggling with difficulty to exclude quinone‐mediated electron exchange between heme *b*_L_ and 2Fe2S that has been predicted to occur under conditions when heme *b*_H_ cannot accept electrons from heme *b*_L_. (i.e., conditions favoring reverse reaction at the Q_i_ site or antimycin inhibition of the Q_i_ site) [43, 44]. Such exchange would reduce the efficiency of the catalytic cycle, leading to energy loss. Therefore, such a process must be somehow minimized or eliminated. In view of the difficulties to explain the molecular basis of EB, one can claim that the mechanistic principles of EB might still be missing. This could be a reason why EB has not been successfully replicated in synthetic systems [45]. It should be noted that the EB reaction described here refers only to the primary proton and electron transfer events and does not encompass the entire catalytic Q cycle, including the conformational movement of the ISP domain.

## The canonical mechanism of electron bifurcation

**Fig. 2:**
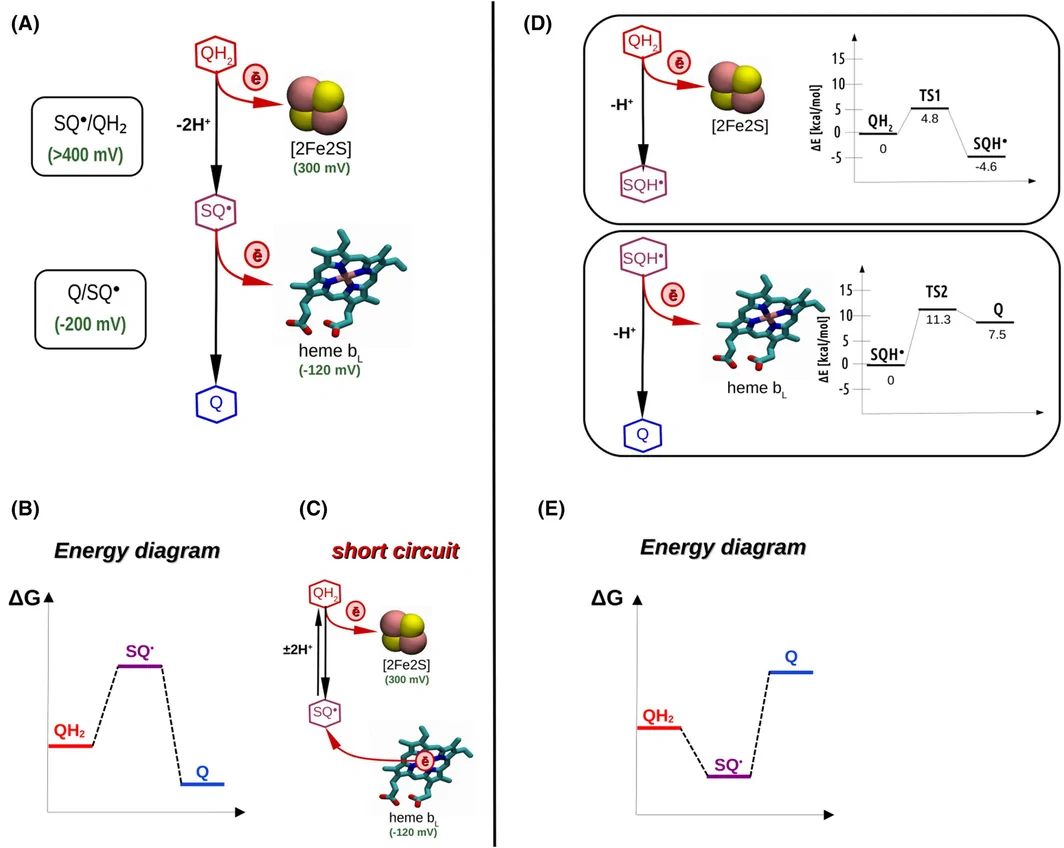
General overview of the canonical QBEB mechanism in Cyt*bc*_*1*_. (A) The two‐step mechanism of quinol (QH_2_) and semiquinone (SQ) oxidation by the Rieske cluster (2Fe2S) and heme *b*_L_, respectively, based on the equilibrium redox midpoint potential (*E*_m_) values. (B) An expected energy diagram postulated in the framework of the canonical quinone‐based electron bifurcation mechanism (QBEB) based on the experimentally determined *E*_m_ values of heme *b*_L_ and 2Fe2S. (C) Predicted short‐circuit reaction based on the canonical QBEB. (D) QM/MM calculations performed with the assumptions derived from the canonical QBEB model, which treat the process as two separate one‐electron reactions reported in [47]. (E) Energy diagram created by combining the QM/MM results obtained for the two reaction steps, modeled with two distinct QM/MM models, which are shown in Figure D.

The original mechanism of EB was based on the formalism of *E*_m_s of the quinone species (the Q/SQ/QH_2_ triad) and their immediate electron acceptors, heme *b*_L_ and 2Fe2S [35, 36, 46]. In this framework, EB is considered as a two‐step mechanism of one‐electron reactions involving the SQ/QH_2_ (semiquinone/quinol) and Q/SQ (quinone/semiquinone) redox couples, which are proposed to differ significantly in their redox midpoint potentials (*E*_m_(SQ/QH_2_) > ~ +400 mV and *E*_m_(Q/SQ) < ~ −200 mV) [11, 46]. The large difference between *E*_m_s of these couples was expected to drive EB at the Q_o_ site. Considering the equilibrium redox midpoint potentials of the electron acceptors at the site: *E*_m_ ~ +300 mV for 2Fe2S and *E*_m_ ~ −120 mV for heme *b*_L_, the 2Fe2S is expected to act as the acceptor of the first electron from QH_2_. Since *E*_m_ of the SQ/QH_2_ couple is larger than *E*_m_ of 2Fe2S and *E*_m_ of the Q/SQ couple is lower than *E*_m_ of heme *b*_L_, it is expected that the first ET step from QH_2_ to 2Fe2S is uphill, while the second ET from the SQ to heme *b*_L_ is downhill. These assumptions constitute the canonical quinone‐based EB mechanism (canonical QBEB) schematically shown in Fig. 2A.

Canonical QBEB has two main consequences. First, the SQ intermediate should be transient and unstable, and the Q/SQ couple, due to a low *E*_m_, can be potentially involved in one‐electron reduction of molecular oxygen to form the superoxide radical [48, 49, 50, 51]. Second, when the Q_i_ site is inhibited, the inability to oxidize hemes *b*_H_ and *b*_L_ should promote ET from heme *b*_L_ to 2Fe2S, mediated by SQ. This reaction, called short circuit (SC, Fig. 2C), leads to uncoupling of the Q_i_ site from the Q_o_ site and maintains QH_2_ oxidation at the Q_o_ site without involvement of the Q_i_ site [43].

## Experimental and theoretical issues of canonical QBEB mechanism

The canonical QBEB mechanism has been widely used in the interpretation of experimental studies, although some difficulties have been encountered. One of them is the mechanism of Cyt*bc*_1_ inhibition by antimycin. In the presence of antimycin, heme *b*_H_ and, transiently, heme *b*_L_ cannot be oxidized by the Q_i_ site. The canonical QBEB predicts that, under these conditions, both these hemes can be reduced by the two consecutive cycles of QH_2_ oxidation taking place at the Q_o_ site, and since their oxidation is blocked, they remain in the reduced state. In the next cycle, the Q_o_ site, 2Fe2S, can still withdraw an electron from QH_2_; however, the resulting SQ will rapidly be reduced to QH_2_ by a downhill reaction of ET from the reduced heme *b*_L_ [43, 46]. As a consequence, the enzyme should catalyze QH_2_ oxidation by ET only through the c‐chain without involvement of the Q_i_ site (SC reaction, Fig. 2C) [43, 44, 46, 52, 53, 54]. Such conditions, favoring energy‐wasting SC reactions, occur not only upon binding of antimycin at the Q_i_ site but also when the Q_i_ site binds QH_2_ [43, 46, 55]. However, reported experiments do not support these expectations and internal SC are not observed [3, 43, 44, 46, 52].

Direct electron transfer (ET) between heme *b*_L_ and the 2Fe2S cluster is unlikely, as the relatively large distance between them (22 Å) makes the process extremely slow [56]. However, the problem appears when a redox‐active molecule such as quinone (QH_2_/SQ/Q) occupies the Q_o_ site and facilitates SC electron transfer [43, 56, 57]. To explain the absence of SC reactions, several ‘gating’ mechanisms were proposed (see the summary of gating mechanisms in [3]). Alterative explanation evoked the concept of ‘concerted’ mechanism, which assumed that during EB electron transfer is not sequential but rather considered as a single step of two‐electron transfer from QH_2_ to heme *b*_L_ and 2Fe2S without formation of SQ [58, 59, 60]. However, the ‘concerted’ mechanism is difficult to reconcile with the detected SQ intermediate at the Q_o_ site and ROS generation by Cyt*bc*_1_ [61, 62, 63, 64, 65, 66, 67, 68].

**Fig. 3:**
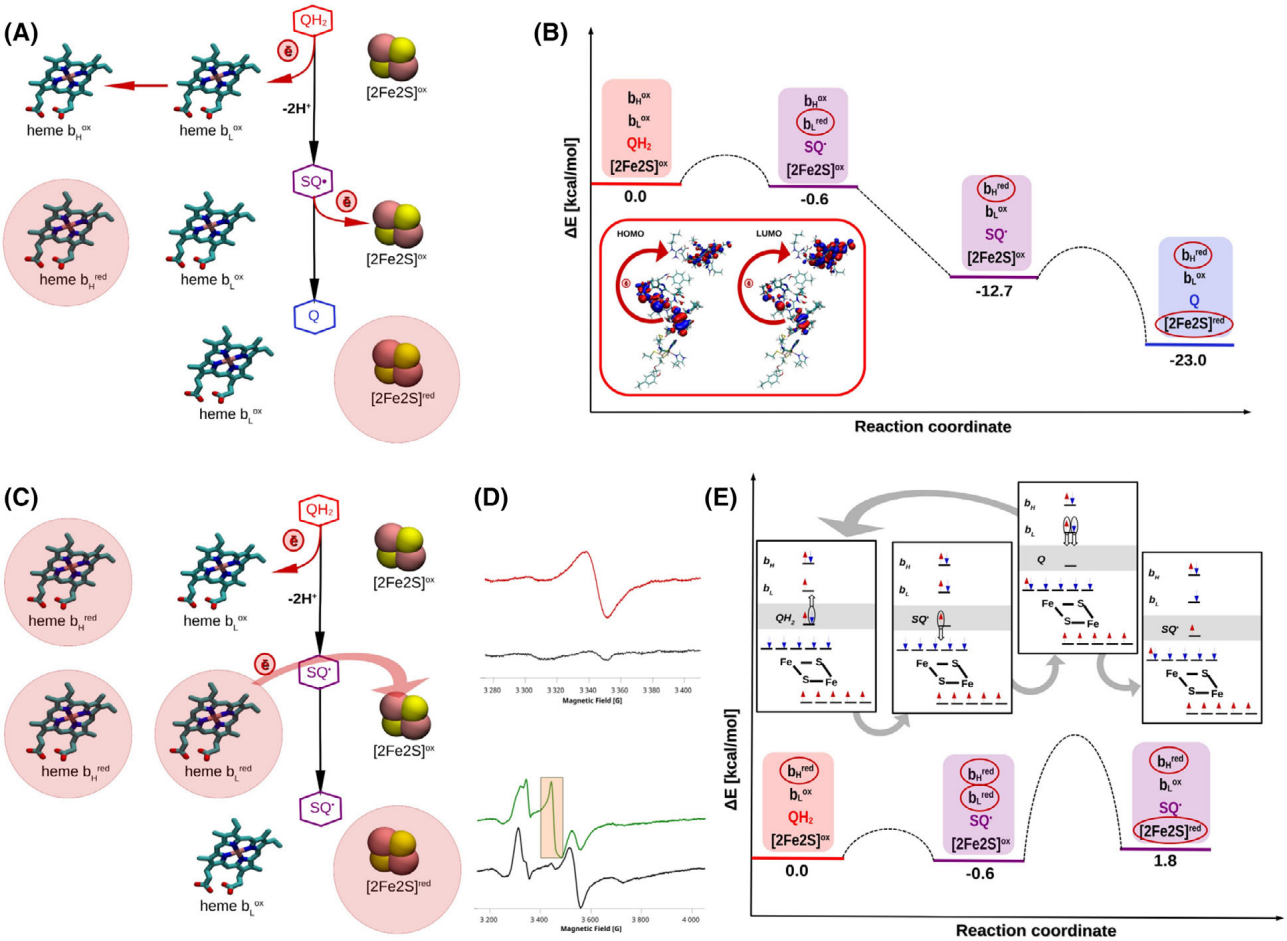
Principles of the EMET mechanism of QH_2_ oxidation in Cyt*bc*_1_ [69]. (A) Simplified scheme of electron transfer (ET) reactions of electron bifurcation (EB) in non‐inhibited enzyme. (B) Simplified energy diagram containing important stationary points involved in the catalytic reaction shown in A. Red inset shows highest occupied and lowest unoccupied molecular orbitals (HOMO and LUMO, respectively), indicating the direction of ET marked with a red arrow. (C) Simplified scheme of ET reactions occurring in the antimycin‐inhibited enzyme. (D) EPR signals of semiquinone (SQ, red) and semiquinone ferromagnetically coupled to reduced 2Fe2S (SQ‐2Fe2S^red^), trapped during the turnover (orange box in green spectrum) [69]. Black spectra show the respective signals obtained for the samples after the reaction. (E) Simplified energy diagram presenting stationary points and their electronic configurations explaining experimentally detected coexistence of SQ‐ 2Fe2S^red^ and *b*_L_^ox^ in the antimycin‐inhibited enzyme.

In 2013, the EPR spectra were reported that revealed two species that can be assigned to SQ at the Q_o_ site. A single line of a free radical SQ at *g* ≈ 2 was detected at 200 K and a new transition at *g* = 1.94 measured at X band, 20 K in the region of the 2Fe2S spectrum (Fig. 3D). The latter signal was clearly distinguishable from the signal at *g* = 1.90 assigned to reduced 2Fe2S (2Fe2S^red^) and highly sensitive to Q_o_ inhibitors. It revealed a rhombic spectrum with g values being frequency dependent, and it was interpreted as spin–spin exchange interaction with an estimated isotropic exchange constant of 3.5 GHz between SQ and 2Fe2S^red^ [63]. Importantly, this signal was detected along with oxidized heme *b*_L_ (*b*_L_^ox^), which was confirmed in other experiments [63, 66, 68]. This result was inconsistent with canonical QBEB, which predicts that in the antimycin‐inhibited enzyme, SQ should be detected along with reduced heme *b*_L_ (*b*_L_^red^).

Further mutagenesis studies revealed that production of the SQ‐2Fe2S^red^ intermediate increases in mutants that facilitate interactions between ISP‐HD and the Q_o_ site (known as +1Ala and + 2Ala) and is not observed in the G167P mutant, which significantly hinders ISP‐HD docking at the Q_o_ site [70]. It was found that SQ‐2Fe2S^red^ does not react with molecular oxygen and thus may constitute protection against ROS production [68]. From the point of view of the enzyme's self‐protection against the destructive influence of stopping the electron flow at the Q_i_ level, the SQ‐2Fe2S^red^ state formation seems to be very favorable. Nevertheless, according to canonical QBEB, the reduction in SQ by b_L_^red^ should be downhill; thus, the expected level of SQ‐2Fe2S^red^ signals should be very low.

The mechanism of EB has been a subject of computational studies. Most of the currently reported quantum mechanical (QM) and combined quantum‐mechanical/molecular‐mechanical (QM/MM) studies focused only on explaining the first step of EB assuming *a priori* the scenario predicted by canonical QBEB, that is, the first ET step as QH_2_ oxidation by 2Fe2S [47, 71, 72, 73, 74, 75]. To our knowledge, the complete EB reaction has been considered only once in the literature using QM/MM studies [47]. In this work, the QM/MM studies were preceded by molecular dynamics (MD) simulations of the highly complex model of III_2_IV_2_ supercomplex from *Mycobacterium smegmatis*. The MD simulations were performed to establish a representative structure of Q_o_‐menaquinol complex for the construction of QM/MM models. Two distinct QM/MM models were developed to investigate consecutive steps of the EB reaction dictated by canonical QBEB theory. The first model (QM1) contained the QM region limited to QH_2_, 2Fe2S^ox^ and some protein residues, while in the second model (QM2), the QM part, besides key amino acids, included only *b*_L_^ox^. Consequently, the QM and MM parts differed between the two models according to the specific reaction being investigated. Hybrid QM/MM umbrella sampling simulations were then performed to obtain free‐energy profiles of the proton‐coupled electron transfer reactions. Importantly, the QM level of theory was used to calculate either the first ET from QH_2_ to 2Fe2S^ox^ leading to 2Fe2S^red^ and SQ without involvement of heme *b*_L_, or the second ET from SQ to *b*_L_^ox^ leading to Q and *b*_L_^red^ without involvement (nor presence in the QM region) of 2Fe2S (Fig. 2D). The reported QM/MM results revealed that the SQ‐containing state is the most stable state among all considered, it is by 4.6 and 7.7 kcal·mol^−1^ more stable than the QH_2_ and Q‐containing states, respectively (Fig. 2E) [47]. Such conclusions are inconsistent with the transient and unstable SQ (compare Fig. 2C with Fig. 2E). We note that in this case, the QM/MM calculations were explicitly based on the assumptions of the canonical QBEB framework, yet they yielded results that contradicted its key predictions. This is a highly valuable result, which, in our view, reveals internal inconsistency of canonical QBEB formalism.

## The emergent feature of the redox system of cofactor in Cyt*bc*_1_

The problems addressed above were motivation to perform QM calculations without a pre‐imposed EB mechanism. This approach required using a large cluster model containing crucial amino acids and both electron acceptors 2Fe2S and heme *b*_L_ directly involved in the QH_2_ oxidation [69]. The QM analysis of such a model identified energetically accessible intermediate states along the catalytic pathway, providing the basis for the formulation of a new EB mechanism.

The HOMO and LUMO orbitals for the optimized Q_o_‐QH_2_ complex for this model indicated that EB is initiated by ET from QH_2_ to *b*_L_^ox^ (Fig. 3B). This result was unexpected as it did not agree with the sequence of electron transfers predicted from *E*_m_s of 2Fe2S and heme *b*_L_ [11, 12, 45]. However, the reaction of ET to heme *b*_L_ was initiated in this model only with both 2Fe2S and heme *b*_L_ present (when the model was deprived of 2Fe2S, ET did not proceed), suggesting that consideration of properties of cofactors as separate entities for the reaction mechanism might not be relevant. Rather, EB should be considered as the result of an emergent feature of interacting redox cofactors. The mechanism incorporating this concept was named EMET (EMergent Electron Transfer) to distinguish it from the canonical QBEB.

According to EMET, the first step of EB is ET from QH_2_ to *b*_L_^ox^, coupled with proton transfer from QH_2_ to a nearby proton acceptor (E295 in Cyt*b* of *R. capsulatus*), and subsequent proton transfer from SQH to the histidine ligand of 2Fe2S (H156 in ISP of *R. capsulatus*) (Fig. 3A,B) [69]. Importantly, ET from QH_2_ to *b*_L_^ox^ takes place only when 2Fe2S^ox^ is present at the Q_o_ site. The further progress of the reaction requires removal of the negative charge of *b*_L_^red^ away from the Q_o_ site. This is accomplished by oxidation of *b*_L_^red^ by *b*_H_^ox^ (Fig. 3). This reaction is coupled to (i) proton transfer from E295 to D278 via H276 (residue numbering corresponds to Cyt*bc*_1_ from *R. capsulatus*) and (ii) electron transfer from SQ to 2Fe2S^ox^ to form low‐energy product Q (Fig. 3AB).

The EMET mechanism assumes a QH_2_ binding mode analogous to stigmatellin and does not predict a change in QH_2_ position during the entire QH_2_ oxidation catalyzed at the Q_o_ site. In this position, QH_2_ interacts via water molecule with E295, providing an efficient proton wire (QH_2_‐H_2_O distance of 1.5 Å; H_2_O‐E295 distance of 1.7 Å). The opposite hydroxyl group of QH_2_ forms hydrogen bond (1.8 Å) with H156 coordinating 2Fe2S [69].

When the Q_i_ site is blocked by antimycin (i.e., heme *b*_H_ is kept reduced), the system evolves to the state containing *b*_L_^ox^ and SQ, ferromagnetically coupled to 2Fe2S^red^, which was previously recognized by EPR studies (Fig. 3D) [63, 66, 67, 68]. The *b*_L_^ox^‐SQ‐2Fe2S^red^ state is formed by electron shuffling that occurs on heme *b*_L_ when ET from heme *b*_L_ to heme *b*_H_ is blocked (Fig. 3E). The formation of *b*_L_^ox^‐SQ‐2Fe2S^red^ state is reversible and the enzyme can easily return to the catalytic path when the Q_i_ site is unblocked. Interestingly, according to EMET mechanism, QH_2_ cannot be oxidized to SQ by 2Fe2S, thus the conditions favoring SQ‐mediated short circuit (ET from *b*_L_^red^ to SQ) do not exist. It means that there is no need to introduce any gating mechanisms to suppress these energy‐wasting reactions. Additionally, EMET explains inhibition of QH_2_ to Q oxidation at the Q_o_ site under steady‐state conditions in the presence of antimycin bound at the Q_i_ site.

An important feature of the EMET mechanism is that the suppression of SC is secured even when the ISP‐HD interacts with Cytb. This means that for EMET there is no need to consider the ISP‐HD movement toward Cytc_1_ as part of the SC suppression mechanism. Such considerations have often been evoked for the canonical QBEB mechanism, which, as discussed, above faces general difficulty in explaining the absence of SC in the catalytic mechanism. From this perspective, EMET is a mechanism that would be highly effective for the suppression of SC in complexes, such as cytochrome bcc [76], where the ISP domain is conformationally fixed in one position and transfers electron toward cytochrome aa_3_ oxidase without the movement characteristic for the cytbc_1_ complexes. However, this hypothesis requires further investigation.

## The role of proton transfer

**Fig. 4:**
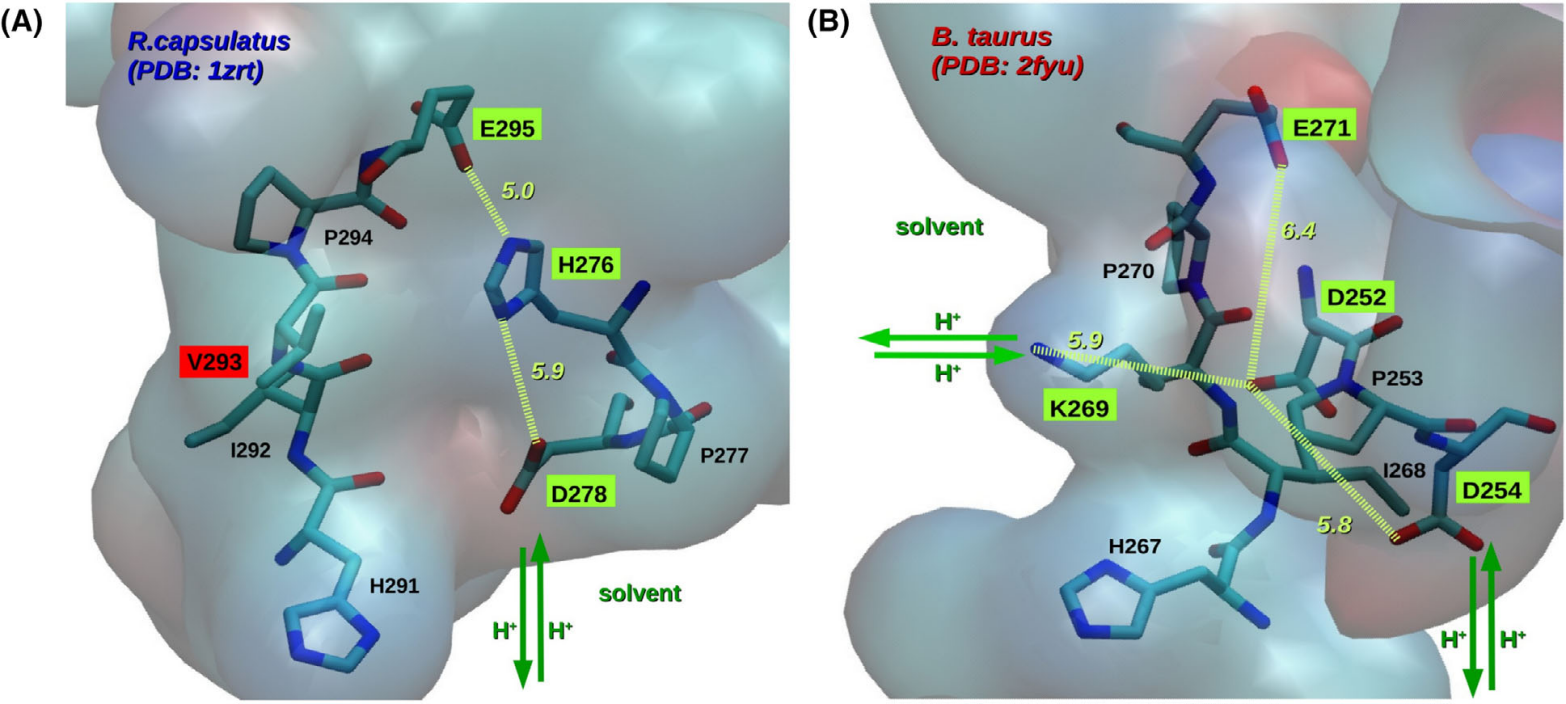
Comparison of proton pathways out of the Q_o_ site in bacterial and mitochondrial quinol oxidation site (Q_o_) to the solvent. (A) Proton single‐path in the structure of bacterial cytochrome *bc*_1_ (Cyt*bc*_1_, PDB code: 1ZRT). (B) Proton double‐path in mitochondrial Cyt*bc*_1_ (PDB code: 2FYU).

It is important to note that the EMET mechanism was developed based on experimental and theoretical studies on Cyt*bc*_1_ from *R. capuslatus*, for which the *b*_L_^ox^‐SQ‐2Fe2S^red^ state has been recognized by EPR as a metastable state under antimycin inhibition [69]. In this case, SQ‐2Fe2S^red^ is formed as result of ET from *b*_L_^red^ to SQ/2Fe2S^ox^. This reaction occurs only when SQ is stabilized by two hydrogen bonds formed by protons derived from QH_2_ and remaining on their direct acceptors (E295 and H156). Dissociation of any of these protons will destabilize SQ in the *b*_L_^red^‐SQ‐2Fe2S^ox^ state leading to *b*_L_^red^‐Q‐2Fe2S^red^. Thus, the energy barriers for the proton transfer reactions at the Q_o_ site will dictate relative levels of both states. In *R. capsulatus* the *b*_L_^ox^‐SQ‐2Fe2S^red^ state is observed because the proton transfer from E295 to D278 via H276 has a large barrier when heme *b*_L_ is reduced (Fig. 4A). However, in enzymes with more efficient proton pathway, the *b*_L_^ox^‐SQ‐2Fe2S^red^ state might not be detectable, as the *b*_L_^red^‐Q‐2Fe2S^red^ state would be dominant. This may be the case of mitochondrial enzyme for which more efficient proton transfers are expected if compared to the bacterial system (Fig. 4B).

## Concluding remarks

The formalism of equilibrium electrochemistry is effective for predicting the overall direction of electron flow in multi‐cofactor redox systems [77]. However, it may not be sufficient for describing electron transfer events in complex multi‐cofactor catalytic sites. This appears to be the case of the Q_o_ site of Cyt*bc*_1_ catalyzing EB. In this site, the substrate is placed between two cofactors, which both accept electrons during catalytic quinol oxidation. The canonical QBEB mechanism proposed the specific sequence of EB, which derived from the assumption that the *E*_m_ value of each redox‐acting pair remains constant during the reaction and can be treated independently. While this sequence of EB steps became widely accepted, canonical QBEB has been having difficulties in explaining several key experimental observations. This suggested that the mechanistic principle of EB might be missing.

When both electron acceptors are included in a single QM model to analyze EB without any *a priori* assumptions about the sequence of ET events, a new EB mechanism (EMET), featuring a novel sequence of ET steps, was revealed. EMET explained straightforwardly key experimental observations that canonical QBEB has been struggling with. EMET revealed EB to be a result of the emergent mode of action of interacting redox cofactors of the Q_o_ site, which all are involved at every step of the reaction. According to EMET, such a system is bound to experience local changes in dielectric properties and protonation states that affect the electron affinity of redox‐active components.

EMET implicates that redox potentials of individual cofactors measured at equilibrium might not be necessarily applicable for description of reactions in multi‐cofactor catalytic sites, such as the Q_o_ site. While EMET offers a new perspective for understanding the mechanism of EB, it still, as a concept, requires further experimental testing. This framework is consistent with well‐established phenomena, such as modulation of the electron affinity of redox‐active cofactors by changes in dielectric properties and protonation states [78]. Cases in which the formalism of equilibrium electrochemistry fails should therefore be taken seriously and investigated more broadly, as they might reveal a fundamental feature of biological systems—their emergent mode of action.

## Conflict of interest

The authors declare no conflict of interest.

## Author contributions

AWA and MS were involved in conceptualization. AWA was involved in writing – original draft. AWA, MS, and AO were involved in writing – review & editing.

## Data Availability

No new data were generated or analyzed in this review. All information discussed is based on previously published literature cited in the manuscript.

## References

[1] Berry EA , Huang L‐S , Saechao LK , Pon NG , Valkova‐Valchanova M and Daldal F (2004) X‐ray structure of *Rhodobacter capsulatus* cytochrome bc1: comparison with its mitochondrial and chloroplast counterparts. Photosynth Res 81, 251–275. 16034531 10.1023/B:PRES.0000036888.18223.0e

[2] Berry EA , and Walker FA (2008) Bis‐histidine‐coordinated hemes in four‐helix bundles: how the geometry of the bundle controls the axial imidazole plane orientations in transmembrane cytochromes of mitochondrial complexes II and III and related proteins. J Biol Inorg Chem 13, 481–498. 18418633 10.1007/s00775-008-0372-9 PMC2805440

[3] Sarewicz M , Pintscher S , Pietras R , Borek A , Bujnowicz Ł , Hanke G , Cramer WA , Finazzi G and Osyczka A (2021) Catalytic reactions and energy conservation in the cytochrome bc1 and b6f complexes of energy‐transducing membranes. Chem Rev 121, 2020–2108. 33464892 10.1021/acs.chemrev.0c00712 PMC7908018

[4] Tso S‐C , Shenoy SK , Quinn BN and Yu L (2000) Subunit IV of cytochrome bc 1 complex from *Rhodobacter sphaeroides* . J Biol Chem 275, 15287–15294. 10748084 10.1074/jbc.M907367199

[5] Berry EA , De Bari H and Huang L‐S (2013) Unanswered questions about the structure of cytochrome bc1 complexes. Biochim Biophys Acta 1827, 1258–1277. 23624176 10.1016/j.bbabio.2013.04.006

[6] Kleinschroth T , Castellani M , Trinh CH , Morgner N , Brutschy B , Ludwig B and Hunte C (2011) X‐ray structure of the dimeric cytochrome bc1 complex from the soil bacterium *Paracoccus denitrificans* at 2.7‐Å resolution. Biochim Biophys Acta 1807, 1606–1615. 21996020 10.1016/j.bbabio.2011.09.017

[7] Kriauciunas A , Yu L , Yu CA , Wynn RM and Knaff DB (1989) The *Rhodospirillum rubrum* cytochrome bc1 complex: pep tide composition, prosthetic group content and quinone binding. BBA‐Bioenergetics 976, 70–76. 2548618 10.1016/s0005-2728(89)80190-2

[8] Koch H‐G , and Schneider D (2007) Folding, assembly, and stability of transmembrane cytochromes. Current Chemical Biology 1, 59–74.

[9] Volkmer T , Becker C , Prodöhl A , Finger C and Schneider D (2006) Assembly of a transmembrane b‐type cytochrome is mainly driven by transmembrane helix interactions. Biochim Biophys Acta 1758, 1815–1822. 16860778 10.1016/j.bbamem.2006.05.009

[10] Xia D , Esser L , Tang W‐K , Zhou F , Zhou Y , Yu L and Yu C‐A (2013) Structural analysis of cytochrome bc1 complexes: implications to the mechanism of function. Biochim Biophys Acta 1827, 1278–1294. 23201476 10.1016/j.bbabio.2012.11.008 PMC3593749

[11] Bergdoll L , Ten Brink F , Nitschke W , Picot D and Baymann F (2016) From low‐ to high‐potential bioenergetic chains: thermodynamic constraints of Q‐cycle function. BBA‐Bioenergetics 1857, 1569–1579. 27328272 10.1016/j.bbabio.2016.06.006

[12] Zhang H , Chobot SE , Osyczka A , Wraight CA , Dutton PL and Moser CC (2008) Quinone and non‐quinone redox couples in complex III. J Bioenerg Biomembr 40, 493–499. 18975063 10.1007/s10863-008-9174-6 PMC2664707

[13] Shinkarev VP and Wraight CA (2007) Intermonomer electron transfer in the bc1 complex dimer is controlled by the energized state and by impaired electron transfer between low and high potential hemes. FEBS Lett 581, 1535–1541. 17399709 10.1016/j.febslet.2007.03.037 PMC1997310

[14] Pintscher S , Wójcik‐Augustyn A , Sarewicz M and Osyczka A (2020) Charge polarization imposed by the binding site facilitates enzymatic redox reactions of quinone. Biochim Biophys Acta Bioenerg 1861, 148216. 32387188 10.1016/j.bbabio.2020.148216

[15] Zhang Z , Huang L‐S , Shulmeister VM , Chi Y‐I , Kim KK , Hung L‐W , Crofts AR , Berry EA and Kim S‐H (1998) Electron transfer by domain movement in cytochrome bc1. Nature 392, 677–684. 9565029 10.1038/33612

[16] Wilson CA and Crofts AR (2018) Dissecting the pattern of proton release from partial process involved in ubihydroquinone oxidation in the Q‐cycle. BBA‐Bioenergetics 1859, 531–543. 29625088 10.1016/j.bbabio.2018.03.016 PMC5955396

[17] Xia D , Yu C‐A , Kim H , Xia J‐Z , Kachurin AM , Zhang L , Yu L and Deisenhofer J (1997) Crystal structure of the cytochrome bc1 complex from bovine heart mitochondria. Science (New York, NY) 277, 60–66. 10.1126/science.277.5322.60 PMC12235523 9204897

[18] Liebl U , Sled V , Brasseur G , Ohnishi T and Daldal F (1997) Conserved nonliganding residues of the *Rhodobacter capsulatus* Rieske iron–sulfur protein of the bc1 complex are essential for protein structure, properties of the [2Fe‐2S] cluster, and communication with the quinone pool. Biochemistry 36, 11675–11684. 9305957 10.1021/bi970776l

[19] Brasseur G , Sled V , Liebl U , Ohnishi T and Daldal F (1997) The amino‐terminal portion of the rieske iron–sulfur protein contributes to the ubihydroquinone oxidation site catalysis of the *Rhodobacter capsulatus* bc1 complex. Biochemistry 36, 11685–11696. 9305958 10.1021/bi970777d

[20] Darrouzet E , Valkova‐Valchanova M , Moser CC , Dutton PL and Daldal F (2000) Uncovering the [2Fe2S] domain movement in cytochrome bc1 and its implications for energy conversion. Proc Natl Acad Sci USA 97, 4567–4572. 10781061 10.1073/pnas.97.9.4567 PMC18273

[21] Millett F and Durham B (2004) Kinetics of electron transfer within cytochrome bc1 and between cytochrome bc1 and cytochrome c. Photosynth Res 82, 1–16. 16228609 10.1023/B:PRES.0000040434.77061.ca

[22] Millett F , Havens J , Rajagukguk S and Durham B (2013) Design and use of photoactive ruthenium complexes to study electron transfer within cytochrome bc1 and from cytochrome bc1 to cytochrome c. Biochim Biophys Acta Bioenerg 1827, 1309–1319. 10.1016/j.bbabio.2012.09.002 PMC3535674 22985600

[23] Xiao K , Engstrom G , Rajagukguk S , Yu C‐A , Yu L , Durham B and Millett F (2003) Effect of famoxadone on photoinduced electron transfer between the iron–sulfur center and cytochrome c1 in the cytochrome bc1 complex. J Biol Chem 278, 11419–11426. 12525495 10.1074/jbc.M211620200

[24] Cooley JW , Lee D‐W and Daldal F (2009) Across membrane communication between the Qo and qi active sites of cytochrome bc1. Biochemistry 48, 1888–1899. 19254042 10.1021/bi802216h PMC2784637

[25] Brugna M , Rodgers S , Schricker A , Montoya G , Kazmeier M , Nitschke W and Sinning I (2000) A spectroscopic method for observing the domain movement of the Rieske iron–sulfur protein. Proc Natl Acad Sci USA 97, 2069–2074. 10681446 10.1073/pnas.030539897 PMC15755

[26] Cooley JW , Roberts AG , Bowman MK , Kramer DM and Daldal F (2004) The raised midpoint potential of the [2Fe2S] cluster of cytochrome bc1 is mediated by both the Qo site occupants and the head domain position of the Fe‐S protein subunit. Biochemistry 43, 2217–2227. 14979718 10.1021/bi035938u

[27] Darrouzet E , Valkova‐Valchanova M and Daldal F (2002) The [2Fe‐2S] cluster Em as an indicator of the iron–sulfur subunit position in the ubihydroquinone oxidation site of the cytochrome bc1 complex. J Biol Chem 277, 3464–3470. 11707448 10.1074/jbc.M107973200

[28] Sarewicz M , Dutka M , Froncisz W and Osyczka A (2009) Magnetic interactions sense changes in distance between heme bL and the iron–sulfur cluster in cytochrome bc1. Biochemistry 48, 5708–5720. 19415898 10.1021/bi900511b PMC2697599

[29] Sarewicz M , Borek A , Cieluch E , Świerczek M and Osyczka A (2010) Discrimination between two possible reaction sequences that create potential risk of generation of deleterious radicals by cytochrome bc1. Implications for the mechanism of superoxide production. Biochim Biophys Acta 1797, 1820–1827. 20637719 10.1016/j.bbabio.2010.07.005 PMC3057645

[30] Gurbiel RJ , Ohnishi T , Robertson DE , Daldal F and Hoffman BM (1991) Q‐band ENDOR spectra of the Rieske protein from *Rhodobacter capsulatus* ubiquinol‐cytochrome c oxidoreductase show two histidines coordinated to the [2Fe‐2S] cluster. Biochemistry 30, 11579–11584. 1660722 10.1021/bi00113a013

[31] Davidson E , Ohnishi T , Atta‐Asafo‐Adjei E and Daldal F (1992) Potential ligands to the [2Fe‐2S] Rieske cluster of the cytochrome bc1 of *Rhodobacter capsulatus* probed by site‐directed mutagenesis. Biochemistry 31, 3342–3351. 1313292 10.1021/bi00128a006

[32] Mason JR and Cammack R (1992) The electron‐transport proteins of hydroxylating bacterial dioxygenases. Annu Rev Microbiol 46, 277–305. 1444257 10.1146/annurev.mi.46.100192.001425

[33] Gurbiel RJ , Doan PE , Gassner GT , Macke TJ , Case DA , Ohnishi T , Fee JA , Ballou DP and Hoffman BM (1996) Active site structure of Rieske‐type proteins: electron nuclear double resonance studies of isotopically labeled phthalate dioxygenase from pseudomonas cepacia and Rieske protein from *Rhodobacter capsulatus* and molecular modeling studies of a Rieske cente. Biochemistry 35, 7834–7845. 8672484 10.1021/bi960380u

[34] Osyczka A , Dutton PL , Moser CC , Darrouzet E and Daldal F (2001) Controlling the functionality of cytochrome c1 redox potentials in the *Rhodobacter capsulatus* bc1 complex through disulfide anchoring of a loop and a β‐branched amino acid near the heme‐ligating methionine. Biochemistry 40, 14547–14556. 11724568 10.1021/bi011630w

[35] Mitchell P (1975) The protonmotive Q cycle: a general formulation. FEBS Lett 59, 137–139. 1227927 10.1016/0014-5793(75)80359-0

[36] Crofts AR , Meinhardt SW , Jones KR and Snozzi M (1983) The role of the quinone pool in the cyclic electron‐transfer chain of *Rhodopseudomonas sphaeroides*: a modified Q‐cycle mechanism. Biochim Biophys Acta 723, 202–218. 21494412 10.1016/0005-2728(83)90120-2 PMC3074349

[37] Tian H , Sadoski R , Zhang L , Yu C‐A , Yu L , Durham B and Millett F (2000) Definition of the interaction domain for cytochrome c on the cytochrome bc1 complex. J Biol Chem 275, 9587–9595. 10734109 10.1074/jbc.275.13.9587

[38] Darrouzet E , Valkova‐Valchanova M and Daldal F (2000) Probing the role of the Fe‐S subunit hinge region during Q_o_ site catalysis in *Rhodobacter capsulatus* bc1 complex. Biochemistry 39, 15475–15483. 11112533 10.1021/bi000750l

[39] Darrouzet E , Moser CC , Dutton PL and Daldal F (2001) Large scale domain movement in cytochrome bc1: a new device for electron transfer in proteins. Trends Biochem Sci 26, 445–451. 11440857 10.1016/s0968-0004(01)01897-7

[40] Sadoski RC , Engstrom G , Tian H , Zhang L , Yu C‐A , Yu L , Durham B and Millett F (2000) Use of a photoactivated ruthenium dimer complex to measure electron transfer between the Rieske iron–sulfur protein and cytochrome c1 in the cytochrome bc1 complex. Biochemistry 39, 4231–4236. 10757970 10.1021/bi000003o

[41] Rajagukguk S , Yang S , Yu C‐A , Yu L , Durham B and Millett F (2007) Effect of mutations in the cytochrome b ef loop on the electron‐transfer reactions of the Rieske iron–sulfur protein in the cytochrome bc1 complex. Biochemistry 46, 1791–1798. 17253777 10.1021/bi062094g PMC2527182

[42] Hunter CN , Daldal F , Thurnauer MC and Beatty JT (2009) The Purple Phototrophic Bacteria. Springer, Dordrecht.

[43] Osyczka A , Moser CC , Daldal F and Dutton PL (2004) Reversible redox energy coupling in electron transfer chains. Nature 427, 607–612. 14961113 10.1038/nature02242

[44] Ransac S and Mazat J‐P (2010) How does antimycin inhibit the bc1 complex? A part‐time twin. BBA‐Bioenergetics 1797, 1849–1857. 20529661 10.1016/j.bbabio.2010.05.014

[45] Yuly JL , Lubner CE , Zhang P , Beratan DN and Peters JW (2019) Electron bifurcation: progress and grand challenges. Chem Commun 55, 11823–11832. 10.1039/c9cc05611d 31515543

[46] Osyczka A , Moser CC and Dutton PL (2005) Fixing the Q cycle. Trends Biochem Sci 30, 176–182. 15817393 10.1016/j.tibs.2005.02.001

[47] Riepl D , Gamiz‐Hernandez AP , Kovalova T , Król SM , Mader SL , Sjöstrand D , Högbom M , Brzezinski P and Kaila VRI (2024) Long‐range charge transfer mechanism of the III2IV2 mycobacterial supercomplex. Nat Commun 15, 5276. 38902248 10.1038/s41467-024-49628-9 PMC11189923

[48] Muller F , Crofts AR and Kramer DM (2002) Multiple Q‐cycle bypass reactions at the Qo site of the cytochrome bc1 complex. Biochemistry 41, 7866–7874. 12069575 10.1021/bi025581e

[49] Cape JL , Bowman MK and Kramer DM (2006) Understanding the cytochrome bc complexes by what they don't do. The Q‐cycle at 30. Trends Plant Sci 11, 46–55. 16352458 10.1016/j.tplants.2005.11.007

[50] Dröse S and Brandt U (2008) The mechanism of mitochondrial superoxide production by the cytochrome bc1 complex. J Biol Chem 283, 21649–21654. 18522938 10.1074/jbc.M803236200

[51] Borek A , Sarewicz M and Osyczka A (2008) Movement of the iron–sulfur head domain of cytochrome bc1 transiently opens the catalytic Q_o_ site for reaction with oxygen. Biochemistry 47, 12365–12370. 18956890 10.1021/bi801207f

[52] Ransac S , Parisey N and Mazat J‐P (2008) The loneliness of the electrons in the bc1 complex. Biochim Biophys Acta 1777, 1053–1059. 18534187 10.1016/j.bbabio.2008.05.003

[53] Springett R (2021) The proton pumping mechanism of the bc complex. Biochim Biophys Acta Bioenerg 1862, 148352. 33338489 10.1016/j.bbabio.2020.148352

[54] Yuly JL , Zhang P , Lubner CE , Peters JW and Beratan DN (2020) Universal free‐energy landscape produces efficient and reversible electron bifurcation. Proc Natl Acad Sci USA 117, 21045–21051. 32801212 10.1073/pnas.2010815117 PMC7474648

[55] Robertson DE , Giangiacomo KM , de Vries S , Moser CC and Dutton PL (1984) Two distinct quinone‐modulated modes of antimycin‐sensitive cytochrome b reduction in the cytochrome bc1 complex. FEBS Lett 178, 343–350. 6096172 10.1016/0014-5793(84)80630-4

[56] Page CC , Moser CC , Chen X and Dutton PL (1999) Natural engineering principles of electron tunnelling in biological oxidation–reduction. Nature 402, 47–52. 10573417 10.1038/46972

[57] Moser CC , Keske JM , Warncke K , Farid RS and Dutton PL (1992) Nature of biological electron transfer. Nature 355, 796–802. 1311417 10.1038/355796a0

[58] Zhu J , Egawa T , Yeh S‐R , Yu L and Yu C‐A (2007) Simultaneous reduction of iron–sulfur protein and cytochrome bL during ubiquinol oxidation in cytochrome bc1 complex. Proc Natl Acad Sci USA 104, 4864–4869. 17360398 10.1073/pnas.0607812104 PMC1829230

[59] Snyder CH , Gutierrez‐Cirlos EB and Trumpower BL (2000) Evidence for a concerted mechanism of ubiquinol oxidation by the cytochrome bc1 complex. J Biol Chem 275, 13535–13541. 10788468 10.1074/jbc.275.18.13535

[60] Trumpower BL (2002) A concerted, alternating sites mechanism of ubiquinol oxidation by the dimeric cytochrome bc1 complex. Biochim Biophys Acta 1555, 166–173. 12206910 10.1016/s0005-2728(02)00273-6

[61] Cape JL , Bowman MK and Kramer DM (2007) A semiquinone intermediate generated at the Qo site of the cytochrome bc1 complex: importance for the Q‐cycle and superoxide production. Proc Natl Acad Sci USA 104, 7887–7892. 17470780 10.1073/pnas.0702621104 PMC1876542

[62] Zhang H , Osyczka A , Dutton PL and Moser CC (2007) Exposing the complex III Qo semiquinone radical. Biochim Biophys Acta 1767, 883–887. 17560537 10.1016/j.bbabio.2007.04.004 PMC3554237

[63] Sarewicz M , Dutka M , Pintscher S and Osyczka A (2013) Triplet state of the semiquinone‐Rieske cluster as an intermediate of electronic bifurcation catalyzed by cytochrome bc1. Biochemistry 52, 6388–6395. 23941428 10.1021/bi400624m PMC3889490

[64] Vennam PR , Fisher N , Krzyaniak MD , Kramer DM and Bowman MK (2013) A caged, destabilized, free radical intermediate in the Q‐cycle. Chembiochem 14, 1745–1753. 24009094 10.1002/cbic.201300265 PMC3951126

[65] Victoria D , Burton R and Crofts AR (2013) Role of the ‐PEWY‐glutamate in catalysis at the Qo‐site of the Cyt bc1 complex. Biochim Biophys Acta Bioenerg 1827, 365–386. 10.1016/j.bbabio.2012.10.012 PMC4059559 23123515

[66] Sarewicz M , Bujnowicz Ł , Bhaduri S , Singh SK , Cramer WA and Osyczka A (2017) Metastable radical state, nonreactive with oxygen, is inherent to catalysis by respiratory and photosynthetic cytochromes bc1/b6f. Proc Natl Acad Sci 114, 1323–1328. 28115711 10.1073/pnas.1618840114 PMC5307432

[67] Sarewicz M , Bujnowicz Ł and Osyczka A (2018) Generation of semiquinone‐[2Fe‐2S] + spin‐coupled center at the Qo site of cytochrome bc1 in redox‐poised, illuminated photosynthetic membranes from *Rhodobacter capsulatus* . Biochimica et Biophysica Acta ‐ Bioenergetics 1859, 145–153. 29180241 10.1016/j.bbabio.2017.11.006

[68] Bujnowicz Ł , Sarewicz M , Borek A , Kuleta P and Osyczka A (2019) Suppression of superoxide production by a spin–spin coupling between semiquinone and the Rieske cluster in cytochrome bc1. FEBS Lett 593, 3–12. 30428128 10.1002/1873-3468.13296

[69] Wójcik‐Augustyn A , Bujnowicz Ł , Osyczka A and Sarewicz M (2026) Electron bifurcation arises from emergent features of multicofactor enzymes. ACS Omega 11, 29892–29905. 42222790 10.1021/acsomega.5c13233 PMC13216931

[70] Borek A , Kuleta P , Ekiert R , Pietras R , Sarewicz M and Osyczka A (2015) Mitochondrial disease‐related mutation G167P in cytochrome b of *Rhodobacter capsulatus* cytochrome bc1 (S151P in human) affects the equilibrium distribution of [2Fe‐2S] cluster and generation of superoxide. J Biol Chem 290, 23781–23792. 26245902 10.1074/jbc.M115.661314 PMC4583038

[71] Barragan AM , Schulten K and Solov'yov IA (2016) Mechanism of the primary charge transfer reaction in the cytochrome bc1 complex. J Phys Chem B 120, 11369–11380. 27661199 10.1021/acs.jpcb.6b07394 PMC5721205

[72] Salo AB , Husen P and Solov'yov IA (2017) Charge transfer at the Q_o_ ‐site of the cytochrome *bc* _1_ complex leads to superoxide production. J Phys Chem B 121, 1771–1782. 27983847 10.1021/acs.jpcb.6b10403

[73] Barragan AM , Soudackov AV , Luthey‐Schulten Z , Hammes‐Schiffer S , Schulten K and Solov'yov IA (2021) Theoretical description of the primary proton‐coupled electron transfer reaction in the cytochrome *bc* _1_ complex. J Am Chem Soc 143, 715–723. 33397104 10.1021/jacs.0c07799 PMC8037953

[74] Barragan AM , Crofts AR , Schulten K and Solov'yov IA (2014) Identification of ubiquinol binding motifs at the Qo‐site of the cytochrome bc1 complex. J Phys Chem B 119, 433–447. 25372183 10.1021/jp510022w PMC4297238

[75] Arantes GM (2025) Redox‐activated proton transfer through a redundant network in the Q_o_ site of cytochrome *bc* _1_ . J Chem Inf Model 65, 2660–2669. 40008618 10.1021/acs.jcim.4c02361 PMC11898062

[76] Mathiyazakan V , Tan EXY , Moraski G , Basak S , Saw W‐G , Pethe K and Grüber G (2026) The mycobacterium abscessus cytochrome bcc:aa3 oxidase structure paves the way for an agent targeting subunit QcrB. Nat Commun 17, 4821. 41932870 10.1038/s41467-026-70805-5 PMC13222863

[77] Blumberger J (2015) Recent advances in the theory and molecular simulation of biological electron transfer reactions. Chem Rev 115, 11191–11238. 26485093 10.1021/acs.chemrev.5b00298

[78] Hardy BJ , Dubiel P , Bungay EL , Rudin M , Williams C , Arthur CJ , Guberman‐Pfeffer MJ , Sofia Oliveira A , Curnow P and Anderson JLR (2024) Delineating redox cooperativity in water‐soluble and membrane multiheme cytochromes through protein design. Protein Sci 33, e5113. 38980168 10.1002/pro.5113 PMC11232281

